# A benchmark study on economic impact of Neem Coated Urea on Indian agriculture

**DOI:** 10.1038/s41598-022-12708-1

**Published:** 2022-05-31

**Authors:** K. B. Ramappa, Vilas Jadhav, A. V. Manjunatha

**Affiliations:** 1 Dr. Rajendra Prasad Central Agricultural University, Pusa, Bihar India; 2 Agricultural Development and Rural Transformation Centre (ADRTC), Institute for Social and Economic Change (ISEC), Bengaluru, Karnataka India

**Keywords:** Plant sciences, Environmental sciences, Chemistry

## Abstract

The policy of mandatory production and distribution of Neem Coated Urea (NCU) was implemented by the Government of India since 2015. In this article, authors have made an attempt to explore the benefits of NCU recognized by the producers of six major crops such as paddy, maize, sugarcane, tur, jute and soybean across six major states viz., Karnataka, Maharashtra, Madhya Pradesh, Bihar, Punjab and Assam. The results reveal that NCU use has contributed positively in terms of increasing the yield levels of main product and by-products, as well as net returns with regard to almost all reference crops however; the extent varies from crop to crop. Moreover, NCU has helped reduce the cost of production by minimizing the cost of urea as well as other fertilizers and pesticides usage. Interestingly, the diversion of urea has stopped completely, post the production and distribution of NCU. Hence, it is concluded that the application of NCU has a positive impact on Indian agriculture, by way of increasing yield levels & returns for the farming community. These results are in line with the PM's vision of doubling farmers’ income by 2022 and Sustainable Development Goals of the Country.

## Introduction

It is a well-recognized fact that Indian agriculture sector growth was completely sluggish or say even declined in the entire first half of the twentieth century^1,2^ during the British colonial period, whereas, this prototype was reversed post-independence (1947). The introduction of major food grains such as cereals and pulses registered a higher growth at 4.13 per cent during the first decade of independence i.e., 1951–52 to 1960–61^3,4^. The expansion of net area sown and an increase in crop yields were among the major contributors to the food grain growth, although the growth rate of agricultural sector declined during the same period. More importantly, India’s agricultural policy was mainly oriented to institutional reforms such as land reforms and promotion of farming through cooperatives and also., India started focussing more on promoting industrialization, especially post the second five-year plan (1956–1957 to 1960–1961) while largely neglecting the agricultural sector.

During the mid-1960s, a consecutive two-year drought resulted in a significant negative growth of agricultural sector, particularly in terms of food grain production and as a result, the country faced a serious shortage of food grains with many going without a single meal per day. Considering the contribution of the agricultural sector to GDP (about 50 per cent), its poor performance affected adversely the Indian economy as a whole, and even the political regime itself. As a result, the country was forced to import as many as 10 million tons of food grains (mainly wheat) from abroad for two succeeding years. This serious economic crisis forced the Government of India revisit its agricultural policy and accordingly, as a corrective measure, the government called for attention to break through technological innovations, even as it decided to import new agricultural technologies from abroad. And it was an opportune twist of fate for India that the mid-1960s was a phase when new seed- fertilizer technology dissemination started in the tropical developing world. In particular, it was fortunately found that wheat High Yield Varieties (HYVs) developed by CIMMYT in Mexico were found to be suitable to Indian climatic conditions, particularly North Indian states such as Punjab, Haryana, Uttar Pradesh and Madhya Pradesh. As a result, the Government of India was able to achieve food self-sufficiency within a short span of time, and today India is the largest food grain producer (285 million tons during 2018–2019) in the world, though with a few ups and downs during the drought years. In this context, chemical fertilizer, particularly NPK fertilizers, played a significant role in enhancing agricultural production.

The application of chemical fertilizers has considerably improved the quantity and quality in terms of plant parameters such as, grain yield, leaf area, plant growth, photosynthesis and ultimately, main product and by-product yields. Thereby, chemical fertilizers have increased food availability and income of the farming community besides ensuring food security of the growing population of the nation today, but the impact of their long-run application is a much-debated issue, especially among environmentalists. Among fertilizers, urea is an essential plant nutrient, being a component of amino acids, nucleic acids, nucleotides, chlorophyll, enzymes, and hormones. Nitrogen (N) is a major plant nutrient directly influencing crop growth and improving grain yield and grain quality through higher tilling, leaf area development, grain formation, grain filling, and protein synthesis. Urea is one of the most widely used sources of nitrogen fertilizer in the world.

Fertiliser consumption in India has been increasing since the beginning of Green Revolution to till date. Similarly, the global fertilizer consumption is likely to cross over 201 million tons by 2019, which is about 25 per cent higher than for the previous year recorded (in 2008). Asia as a whole is the largest consumer of fertilizer in the world and is mostly dependent on the import of all the three major nutrients. India ranks second in terms of fertilizer consumption and third-highest in terms of irrigated area (36.80%) in the world. Presently, Indian fertilizer consumption has jumped from less than a million tonne in the mid-1960s to 27 million tonnes in 2019–2020. Since the 1970s, urea has been a major source of nitrogen fertilizer, which is about 83 per cent of the total consumption. Thus, India has emerged as the second largest consumer of urea in the world.

The consumption of urea has slightly increased from 16.95 million tonnes in 2014–2015 to 17.63 million tonnes in 2019–20. With a record urea production of 24.5 MT in 2015–2016, its dominance as a major source of nitrogen fertilizer in India is likely to continue in the future as well^5,6^. Further, fertilizer production shows a mixed growth over the previous year. The production of Urea and NP/NPKs has declined, while that of DAP and SSP has recorded an increase. The production of Urea and NP/NPKs has declined by 0.5% and 3.5%, respectively, during 2019 over 2018. Conversely, the production of DAP has recorded a sharp increase of 21.9 per cent, followed by a moderate increase of 2.8 per cent in the case of SSP during the same period^7^. The decline in urea consumption and an increase in complex nutrient are most likely due to a greater awareness level and advice on the large savings in fertilizer costs, increased efficiency and income afforded by nutrient management planning on farms.

Per hectare consumption of NPK amounts to 128.02 kg/ha in India. Moreover, NPK consumption ratio, which was 6.10:2.46:1, during the period 2014–15, has increased to 7.23:2.9:1 in the year 2019, as against an ideal NPK (Nitrogen-Phosphorus-Potassium) consumption ratio of 4:2:1. This confirms that a marginal improvement in the consumption of fertilizer ratios since 2014–2015 due to the introduction of Neem Coated Urea (NCU) by the Central Government with a view to improving soil health by way of checking an excessive use of (Normal) Urea (NU) has made only a limited headway in the past five years. It shows that there is a further scope for changing the usage pattern of chemical fertilizers for improving soil health. Crops like, maize, wheat and rice (cereals) are the three main fertilizer-consuming crops with their consumption proportion being relatively the same (i.e., 14–16% each)^8–10^. Conversely, a rise in fertilizer consumption over the years, along with the adoption of HYVs and the expansion of groundwater irrigation facilities, has resulted in highest food grain production in India. But the pattern of fertilizer use remains distorted with a growing imbalance in the use of chemical fertilizers, leading to nutrient deficiencies in soils, and thereby deficiency symptoms in plants. Further, there is a huge variation in fertilizer application across various states in India^11–13^.

Considering all the pros and cons of agricultural production, input requirements and urea related subsidy burden, the Government of India has taken a decision of mandatory production and distribution of Neem Coated Urea (NCU) in place of Normal Urea (NU) across the country as a model to follow to create responsible and sustainable strategies. A negligible quantity of neem (neem oil) as a bio-based material is used to coat on urea, thus it reduces the impact on other eco-systems as compared to the NU. The benefits of NCU includes—reduction in the use and release of harmful chemicals; healthy and safety; reduces the quantity of usage of urea, other fertilizers and other plant protection chemicals; controls nematodes, termites, pests and insects; regenerate the soil; improve biodiversity; degrades soil fertility; reduction in cost of cultivation; action of energy efficiency; stops illegal diversion of urea to industry, etc.^14^. In this way, the NCU has more positive impact, in addition to having an ethical and responsible improvement on both the production and consumption sides. Thereby, the role of NCU unquestionably characterizes the lives of all living beings and identifies a potential circular premium^15^. As a tree, neem produces several useful fuels. The quantity of usage of neem oil emulsion in coating urea is very negligible (0.5–1 kg/tonne of urea)^16^ hence, its usage in NCU production may not create a nexus between its usage in food and fuel production. The rise of the bio economy is usually associated with increased sustainability. It is also found that the emission of nitrous oxide is also brought down significantly. Under this background, the GoI has made it mandatory to use NCU and saved Rs. 450 million for the Government from fertilizer subsidy^17^ and make the agriculture sector climate-friendly^18^. In this way, NCU emerged as a bio-based product helped to address issues related to environmental care and focus on sustainable economy. In fact, it has created job opportunities for the landless labourers in the country, and maximised production through increased productivity.

The Ministry of Agriculture and Farmers Welfare (MoA & FW), Government of India, included NCU in the Fertilizer Control Order (FCO) since July 2004, and subsequently made the production and distribution of NCU mandatory from 25th May, 2015. Further, the Government of India launched a Soil Health Card Scheme (SHCS) on 19th February, 2015, with a focussed attention on improving soil quality through a judicious use of fertilizers. Against this background, an attempt has been made by the authors to address the following objectives as part of a benchmark study regarding the impact of NCU on production and productivity of major crops, and its associated benefits to the farming community:

1. To study the trends in usage and pricing of NU and NCU.
2. To explore the adoption behaviour of NCU farmers.
3. To analyse the impact of NCU on yield levels of major crops and income of farmers.

The increased trend in the consumption of urea fertilizer over time is mainly due to a lower cost of NU fertilizer, easy soluble and easy decomposable nature of urea fertilizer in water, relative to other fertilizers available in the market. The characteristics of the NCU are as follows:

1. When prilled (normal) urea is applied to the soil, amide form of urea is rapidly converted to ammonical nitrogen and subsequently to nitrite and nitrate form by the action of nitrifying bacteria viz. Nitrosomonas species and Nitrobacter species, respectively.
2. NCU help to curtail Nitrogen loss besides being absorbed by plants due to volatilization, leaching, runoff and denitrification, causing a serious disruption in ecosystem, soil and health.
3. NCU when applied to the soil, the neem triterpenes inhibit the activity of nitrifying bacteria, resulting in delayed transformation of ammonical nitrogen (NH4 + N) into nitrite nitrogen (NO3–N). Therefore, coating of urea with neem cake also acts as a physical barrier, slowing down the process of solubility and volatility due to its anti-bacterial process.
4. Nitrification inhibitory properties of neem cake are responsible for higher exchangeable soil NH4 + N but their effectiveness depends on type of soil, crop and place. Neem based products had significantly lower NO2-N compared to prilled urea.
5. Neem cake has acidic properties which also inhibit the loss of ammonia volatilization through reducing alkalinity of the soil medium. NCU increased concentration of NH4 + N in soil at all growth intervals as compared to NU and it also has lower amount of NO2-N in flood water than prilled urea.
6. NCU also recorded the maximum amount of available nitrogen, phosphorous and potassium (NPK) in soil, it might be due to organic nature and nitrification inhibiting properties and acidic behaviour of neem cake which helped to maintain higher amount of organic carbon and available NPK in soil.
7. NCU urea has gained popularity because of its increased productivity and the increased demand. NCU is required less in quantity with same field size and gives higher crop yields than NU. Although, NCU is not suitable for industrial use, so chances of its illegal diversion to industries will also be lesser. Use of NCU also reduces the carbon footprint and would be more environment-friendly.

## Methodology

### Sampling and data base

In order to explore the impact of NCU on production, productivity and income across selected states of India, the study adopted a very relevant and detailed primary survey-based approach regarding the adoption of NCU. The states and crops selected for the study were based on the major crops (in terms of area) in each state. The study covered six very important cropping systems. Paddy, Tur, Sugarcane, Maize, Soybean and Jute. The states included Assam, Bihar, Karnataka, Maharashtra, Madhya Pradesh and Punjab. A total of 50 farmers from each taluk were selected, adding up to 100 farmers from each district. Thus, a total of 200 farmers for each crop were interviewed from states. Households were selected randomly for assessing the NCU fertilizer use and its impact on crop production. Further, households were post-classified into two categories – NCU (Neem Coated Urea) users and non-users (those using Normal Urea)—mainly to examine the impact of NCU, as compared to NU. To make the coverage exhaustive, a total of roughly 1200 sample farmers were surveyed (800 neem coated urea (NUC) farmers and 400 normal urea farmers (NU). The reference period of the study was Kharif season for the agriculture year 2015. Both irrigated and rainfed crops accounting for highest urea under cultivation from each of the selected states were considered for the study. For each crop, two districts were selected based on the area under the selected crop and their urea usage within the state. From each district, two taluks/tehsils were selected based on the same criterion. Within the selected taluks, two clusters comprising three to four villages per cluster were selected for conducting the survey.

Further, an adequate care was taken to ensure that the selected crops were grown under chosen irrigated/un-irrigated conditions in the states. The primary data was collected using a pretested structured questionnaire. An adequate care was taken in the selection of representative samples, based on the operational land holding size. The data related to production, import, consumption and prices of urea were also collected from various secondary sources such as, Directorate of economics and Statistics, Indiastat.com, Fertilizers Association of India, Department of Fertilizers (Ministry of Chemicals and Fertilizers), Government of India, and Department of agriculture, cooperation and farmers welfare etc. The details of sample selection are presented in Table 1.

**Table 1:** State-wise, crop coverage and sample size. Source: Authors’ estimates using field survey data.

SI. No./region	Crops	Irrigated/un irrigated	Sample farmers	Total
**South**	**Karnataka**			
1	Paddy	Irrigated	200	**400**
2	Tur	Un-irrigated	200	
**West**	**Maharashtra**			
3	Sugarcane	Irrigated	200	**400**
4	Tur	Un-Irrigated	200	
**Central**	**Madhya Pradesh**			
5	Paddy	Irrigated	200	**400**
6	Soybean	Un-Irrigated	200	
**East**	**Bihar**			
7	Paddy	Irrigated	200	**400**
8	Maize	Un-Irrigated	200	
**North**	**Punjab**			
9	Paddy	Irrigated	200	200
**North-east**	**Assam**			
10	Paddy	Irrigated	**200**	**400**
11	Jute	Un-irrigated	**200**	
**All India**		Irrigated	1200	**2200**

### Analytical tools and techniques

The information gathered from both the primary and secondary sources was analysed, using tabular analysis and Compound Annual Growth Rate (CAGR) exponential functions. CAGR was computed to determine the growth in consumption or sales of urea over time. By taking urea consumption/ sales as a dependent variable (Y_t_) and number of years (t) as independent variable, the exponential function was fitted to computing growth rates. The exponential functional form is as follows.

1 $${\text{Y}} = {\text{ AB}}^{{\text{t}}}$$

Y = Dependent variable (Consumption/sales of urea in the tth year ). A = Constant, B = regression coefficient/ simple growth rate, t = independent variable (time variable i.e., t = 1,2,…0.10).

Applying logarithm on both sides, the equation takes a linear form:

2 $${\text{log Y}} = {\text{ LogA}} + {\text{XLogB}}$$

on writing LogA = a, LogB = b and LogY = y

3 $${\text{The equation becomes y}} = {\text{ a}} + {\text{b}}^{{\text{t}}}$$

4 $${\text{The compound growth rate }}\left( {\text{r}} \right){\text{ is }} = \, \left( {{\text{B}} - {1}} \right) \times { 1}00$$

It is expressed in percentage (%).

In addition, partial budgeting framework and paired unequal sample ‘t’ *test* (between NCU and Non-NCU farmers for the year 2015) were also used for observing the significant differences between the two categories of farmers with respect to various indicators. A partial budgeting technique was employed for comparing the costs and returns related to NCU usage and the potential changes in crop production. Partial budget was divided into three main sections: (I) costs; (II) benefits; and (III) analysis. The analysis section included the net change in profits and break-even analysis (Benefit/Cost Ratio (BCR)). The possible changes occurring in the NCU intervention fell into four categories viz., added returns, reduced returns, added costs, and reduced costs. As part of a partial budgeting, an attempt has been made to present and discuss the comparative figures of costs incurred and returns realized related to NCU usage. Further, a Propensity Score Matching (PSM) technique was applied for estimating the causal treatment (neem coated urea) effects, which is widely used in impact estimation studies^19–22^.

Focussing on seven outcome economic variables such as Age (year), Education (years of schooling), net operated area (acres), ratio of selected crop area to net operated area (%), experience in farming (No. of years), ratio of neem coated urea cost to total fertilizer cost (%) and net income (Rs/acre), PSM was adopted for estimating the predicted probability that a farmer has applied NCU. This probability is also known as the propensity score, which was obtained through the logit model. We have used the standard logit model by assigning values (1 = NCU users and 0 = NU users) to obtain the propensity score, using the methodology as adopted by^23^, The PSM is estimated by using the formula as follows

$${\text{pr}}\left( {{\text{X}}_{{\text{i}}} } \right) \, = {\text{ P}}({\text{Z}} = {1}/{\text{ X}}_{{{\text{i}})}}$$

where pr(X_i_) is the propensity score of the *ith* individual; P(Z = 1/ X_i)_ is the probability of treatment (neem coated urea) given the observable covariates( X) of *ith* individual. A balancing test was conducted to make sure that the difference in covariates of the two groups in the matched sample had been eliminated. For this^24^, used the Mean Absolute Standardized Bias (MASB) between the treated and control group (which should be not more than 20%). According to^25^ compares Pseudo R^2^ and p-values of the likelihood ratio test of the joint insignificance of all repressors’ obtained from the logit model before and after the matching. Particularly, after matching, there should be no systematic difference in the distribution of covariates of the two groups (NUC and NU). Hence, the Pseudo R^2^ or p-values of the likelihood ratio should be insignificant. Several combinations of covariates were tried, in addition to higher order and interaction terms, for a balancing test. However, only the combination of Age, Education, net operated area, ratio of selected crop area to net operated area, experience in farming, ratio of neem coated urea cost to total fertilizer cost, and net income satisfied the test; hence, these covariates were selected at the aggregated level for obtaining the propensity score. In this article, we have employed three matching algorithms viz., NNM (Nearest Neighbour Matching, KBM (Kernel Based Matching) (0.01) and CM (Calliper Matching) (0.01). The average treatment effects on treated with neem coated urea (ATTNCU) are computed by restricting the matches to households with propensity scores that fall in the area of common support:

$${\text{ATTNCU}} = {\text{E }}\left( {{\text{Y}}_{{\text{i}}}^{{1}} {-}{\text{Y}}_{{\text{i}}}^{0} } \right)$$

where ATTNCU is the average treatment effects on neem coated urea treated farmers; E(Y_i_) is the expected value of the impact variable; 1 represents neem coated urea, 0 otherwise.

### Plant ethics statement

"Authors have not used or recommended any plant or specimens, as data were collected from farmers who have been cultivating these crops in their farms over a long period."

### Human ethics

"As a Social Science study, the questionnaires were approved by the Ethics Committee of Institute for Social and Economic Change (ISEC) on the Project entitled "Impact of Neem Coated Urea (NCU) on Production, Productivity and Soil Health in India", which was used as a tool to gather information from farmers. "All experimental protocols were approved by a Research Programmes Committee of ISEC and all methods were performed in accordance with the relevant guidelines and regulations". "The information was collected from the heads of family, who were above 18 years and their 'informed consent' was obtained from all subjects".

## Results and discussion

### Trends in all India urea production, import, consumption, and prices

To spot a pattern, trend analysis is the most common practice; hence, the authors have adopted trend analysis for understanding the growth pattern in terms of production, import, consumption, and prices of urea at the macro level. This technique is often used in extracting an underlying behavioural pattern based on a time-series data, which remains partly or wholly hidden by noise. This method helps understand how, and why, things have changed or are likely to change over time and estimation has been done using a simple or multiple regression analysis. Urea has a high nitrogen content (46%), can adapt to almost all types of soil, and is widely used in the agricultural sector both as a fertilizer and animal feed additive and hence, urea is the most important nitrogenous fertilizer. Considering all these potential benefits, urea is the king of fertilizers.

To spot a pattern, trend analysis is the most common practice; hence, the authors have adopted trend analysis for understanding the growth pattern in terms of production, import, consumption, and prices of urea at the macro level. This technique is often used in extracting an underlying behavioural pattern based on a time-series data, which remains partly or wholly hidden by noise. This method helps understand how, and why, things have changed or are likely to change over time and estimation has been done using a simple or multiple regression analysis. Urea has a high nitrogen content (46%), can adapt to almost all types of soil, and is widely used in the agricultural sector both as a fertilizer and animal feed additive and hence, urea is the most important nitrogenous fertilizer. Considering all these potential benefits, urea is the king of fertilizers.

**Figure 1:**
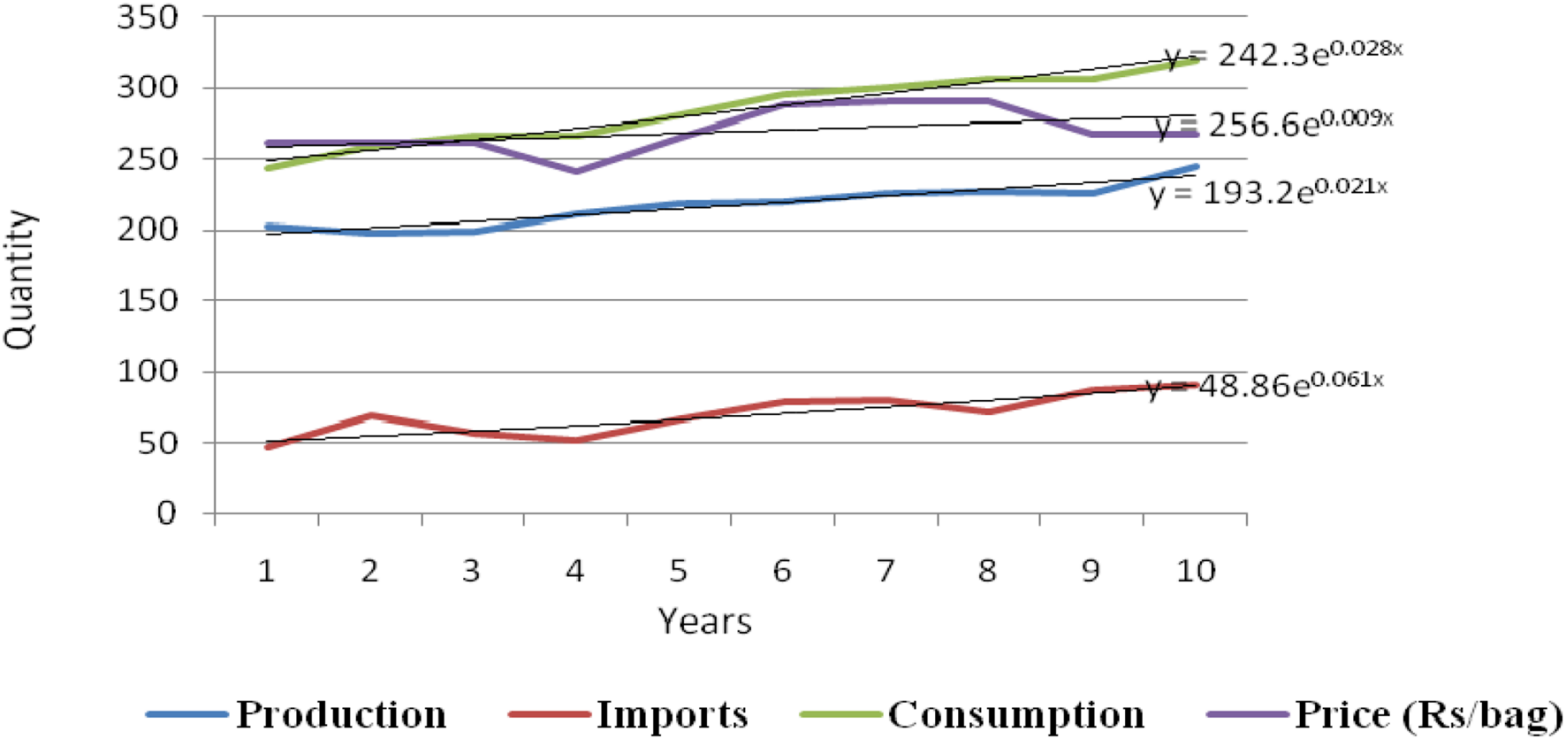
All India urea production, imports, consumption and prices. Source: Authors’ estimates using field survey data.

The state-wise trends in consumption/sale of urea are presented in Table 2 and Fig. 1. It is revealed from Table 2 that the consumption of urea has increased from 8542.92 thousand Metric Tonnes (mt) in 2006–2007 to 11,376.76 thousand mts in 2015–16 at the all India level with a growth rate of 2.94 per cent, and is found statistically significant at five per cent level. From among the selected states, Assam accounts for highest growth rate of (5.50%), followed by Madhya Pradesh (5.10%), Karnataka (2.92%), Maharashtra (2.13%), Punjab (1.92%), and Bihar for the least (1.81%), respectively.

**Table 2:** Trends in all India urea production, import, consumption and prices. (000 mt). Source: Authors’ estimates using indiastat.com data.

Years	Assam	Bihar	Karnataka	Maharashtra	MP	Punjab	All India
2006	194.10	1598.10	1097.58	1985.00	1297.00	2371.14	8542.92
2007	195.41	1851.72	1253.63	2131.00	1425.00	2646.44	9503.20
2008	223.48	1794.82	1281.99	2258.00	1371.00	2576.90	9506.19
2009	251.31	1701.11	1377.07	2289.00	1603.00	2445.76	9667.25
2010	256.61	1691.21	1427.71	2538.00	1669.00	2720.44	10,302.97
2011	304.61	1811.51	1444.80	2481.00	1788.00	2825.70	10,655.62
2012	278.93	2095.96	1446.32	2332.00	1856.00	2842.97	10,852.18
2013	281.51	1870.64	1479.20	2655.00	2224.00	2619.32	11,129.67
2014	299.53	1940.41	1532.60	2572.00	2017.00	2734.26	11,095.80
2015	392.39	1945.52	1462.80	2300.00	2190.00	3086.05	11,376.76
CAGR	5.50*	1.81*	2.92**	2.13**	5.10**	1.92*	2.94**
% change over from 2006–07 to 2015–16	102.16	21.73	33.27	13.69	68.85	30.15	33.17

**^&^*denotes significance level at 1 and 5%, respectively. Figures in parentheses indicate Metric Tonnes (mt).

The percentage change over 2006–2007 to 2015–2016 has been estimated using formula as follows. Percentage change = (New values-Old values/ Old values)*100. The percentage change over (2006–07) values to the existing values also reflect that highest changeover has occurred in respect of Assam (102.16%), followed by Madhya Pradesh (68.85%), Karnataka (33.27%), Punjab (30.15%), Bihar (21.73%) and Maharashtra (13.69%). It is clear from the table that the changeover observed in consumption/ sales in the case of urea amounts to 33.17 per cent (Table 2) at the aggregate level.

### Socio-economic characteristics of the sample households

The general characteristics of the overall sample farmers are shown in Table 3. The table reveals wide variations in the socio-economic characteristics of farmers across the sample states and different crops. The average age of sample farmers across all the states works out to 46 years with a majority of them being male respondents. On an average, the sample farmer family consists of seven members out of which, three have been engaged in farming for the last 24 years. These characteristics are relatively common for all the respondents growing different crops and across States. Coming to literacy level, most (28%) of them have completed Pre-University and above, followed by primary schooling (23%), higher primary (21%) and matriculation (16%); however, about 12 per cent of them are found illiterate. Across crops, a majority (> 60%) of paddy, sugarcane and maize farmers have completed matriculation and above, while in respect of tur, soybean and jute, more than half of the farmers have studied up to higher primary level. With respect to social composition, overall, a majority of the sample farmers belong to general category (51%), followed by Other Backward Classes (OBCs) (29%), Scheduled Castes (11%) and Scheduled Tribes (7%), while in the case of maize, more than half of the farmers (58%) belong to OBC category. The proportion remains relatively the same in respect of all the crops.

**Table 3:** Crop- wise socio-economic characteristics of the sample households. Source: Authors’ estimates using field survey data.

Sl. no.	Particulars	Paddy	Tur	Sugarcane	Maize	**Soybean**	**Jute**	**Overall**
1	Age of respondents (years)	46	48	47	49	45	44	46
2	Male respondents (%)	99.30	98.00	99.00	100.00	95.00	100.00	98.50
3	Family members engaged in farming (no.)	3	3	3	2	4	2	3
4	Experience in farming (years)	23	27	28	19	25	22	24
5	Family size (no.)	7	7	7	7	7	6	7
6	**Literacy level (% farmers)**							
I	Illiterates	9.40	12.25	2.00	-	25.50	20.50	11.60
Ii	Primary (1–4)	8.20	29.00	7.50	21.50	23.50	48.50	23.03
Iii	Higher primary (5–9)	19.10	15.50	19.00	18.00	38.00	17.00	21.10
Iv	Matriculation (10)	19.70	19.75	26.50	19.50	06.50	6.00	16.32
V	Pre-university (10 + 2) and above	43.60	23.50	45.00	41.00	06.50	8.00	27.95
7	**Caste (% farmers)**							
I	General	51.90	43.75	72.50	25.50	11.00	98.90	50.59
Ii	OBC	37.70	34.25	14.00	58.50	30.00	01.10	29.25
Iii	SC	5.80	8.50	4.00	8.50	37.50	–	10.71
Iv	ST	4.60	6.25	0.50	7.50	21.50	–	6.75
V	Others	–	7.25	9.00	–	–	–	2.70

Selected study sates.

### Average size of operational landholding of the sample farmers

The details of operational landholdings of the sample farmers are presented in Table 4. The table reveals that the average net operational area in the study region is comparatively higher in the case of sugarcane farmers (12.6 acres), followed by paddy farmers (10.80 acres) and tur farmers (10.34 acres). The least net operational area (6.37 acres) is observed for jute farmers. While in respect of the remaining cases such as soybean and maize, the net operational area works out to less than 10 acres. As usual, highest operational landholding rests with large farmers only in respect of all the crops, however, it is found to be as high as 27 acres in the case of sugarcane farmers, followed by maize (20.80 acre) and tur farmers (19.25 acres). Overall, a substantial proportion of the operational area is accounted for by land owners (> 7 acres) across all the crops. Interestingly, the leased-in area seems to be highest in respect of paddy crop only (2.25 acres), whereas the proportion is less than an acre/household in respect of other crops. On the contrary, the leased-out area constitutes less than an acre across crops. However, it is highest (0.75 acre) in respect of maize crop for Bihar. Similarly, the proportion of uncultivated or fallow land is found negligible across the sample farmers in the study area. With regard to irrigation, in addition to the irrigated crops such as paddy and sugarcane, soybean also accounts for a major area (> 90% each) under irrigation from among the sample crops, and the remaining area comes under rainfed conditions. Additionally, the area occupied by maize and jute crops under irrigation also constitutes more than 72 per cent. Whereas, tur is majorly grown under rainfed conditions in the states of Karnataka and Maharashtra and hence, the proportion of rainfed area is more (71%). However, about 29 per cent of the farmers grow tur under irrigated conditions as well. It is noted that across categories of farmers, a higher proportion of irrigated land is accounted for by small farmers, followed by medium and large farmers in respect of almost all the irrigated crops.

**Table 4:** Crop-wise, average size of operational landholdings of the sample farmers (Acres). Source: Authors’ estimates using field survey data.

SI. No	Particulars	Paddy	Tur	Sugarcane	Maize	Soybean	Jute	Averages
1	Own land	8.81	10.41	12.78	10.42	7.72	7.07	9.54
2	Uncultivated/ Fallow	0.11	0.28	0.19	0.02	0.1	0.23	0.16
3	Leased-in	2.25	0.21	0.06	–	0.58	0.21	0.66
4	Leased-out	0.53	–	–	0.75	0.01	0.68	0.49
5	Net Operational Area (1–2 + 3–4)	10.80	10.34	12.66	9.70	8.19	6.37	9.68
6	% of irrigated land	90.22	29.35	90.21	78.54	93.59	80.79	77.12
7	% of un irrigated land	9.77	70.65	9.79	21.45	6.41	19.21	22.88
8	Rental value of leased-in land (Rs/Acre)	15,231	3755	4080	5412	13,639	5696	7968.83
9	Rental value of leased-out land (Rs/Acre)	11,917	1917	25,000	5973	11,286	8211	10,717.33

Selected study sates.

The average rental value of leased-in land amounts to a maximum of Rs.15,231/ acre for paddy, followed by soybean (Rs.13,639/ acre) and jute (Rs. 5,696/ acre), while it is less than Rs. 5,000/ acre in the case of tur and sugarcane crops. At the same time, across categories, the rental value of leased-in land is highest (Rs.18, 507/acre) for small farmers, followed by medium farmers (Rs. 14,810/ acre) in respect of paddy, whereas, it is medium farmers (Rs. 14,417/acre), followed by small farmers (Rs. 13,300/ acre) in the case of soybean crop. Relatively, the same situation prevails across other crops. On the other hand, the rental value of leased-out land is slightly less than the value of leased-in land in the sample area. Similar to the rental value of leased-in land, the leased-out land value is highest in respect of paddy (Rs. 11,916/ acre), followed by soybean (Rs. 11,286/ acre). However, the leased-out land value of small farmers in the case of paddy is highest (Rs. 20,294/ acre), as compared to the rental values of leased-in and leased-out land across all crops. Interestingly, no sample farmers are found engaged in leasing-in and leasing-out activities in respect of maize and sugarcane crops, respectively.

### Cropping pattern adopted by the sample farmers in the study area

Given the land resources, agricultural production and profitability can be increased through adoption of a scientific cropping pattern. The adoption of technology in the cropping system depends on many factors such as physical and socio-economic resources, available or made available, when they are needed most. In this background, the cropping patterns followed by different categories of farm households are presented in Table 5.

**Table 5:** Crop-wise, Cropping Pattern adopted by the sample farmers in the study area (%). Source: Authors’ estimates using field survey data.

SI. No	Particulars	Paddy	Tur	Sugarcane	Maize	Soybean	Jute
**I**	**Cereals**						
**1**	Paddy	68.95	9.20	12.90	19.01	4.23	18.94
**2**	Maize	5.34			67.90		
**3**	Basmati	4.72					
	**Sub Total**	**79.01**	**9.20**	**12.90**	**86.91**	**4.23**	**18.94**
**II**	**Pulses**						
**4**	Redgram (Tur)		33.25	1.07			
**5**	Pulses (other than tur)		2.49				
**6**	**Sub Total**	**0.00**	**35.74**	**1.07**	**0.00**	**0.00**	**0.00**
**III**	**Oilseeds**						
**7**	Soya bean		20.00	12.03	12.93	76.94	
**8**	Other Oilseeds		10.56				
	**Sub Total**	**0.00**	**30.56**	**12.03**	**12.93**	**76.94**	**0.00**
**IV**	**Commercial Crops**						
**9**	Sugarcane	2.07		59.40			
**10**	Cotton	7.02	8.33	2.53		18.82	
**11**	Jute						74.79
	**Sub Total**	**9.09**	**8.33**	**61.93**	**0.00**	**18.82**	**74.79**
**V**	Horticultural and fodder crops						
**12**	Onion		3.56	6.02			
**13**	Vegetables	1.14		6.00			6.27
**14**	Fodder	7.23					
**15**	Others	4.22	12.00				
	**Sub Total**	**12.59**	**15.56**	**12.02**	**0.00**	**0.00**	**6.27**
**VI**	Gross cropped area (%)	101	99	100	100	100	100

Paddy-farmers across the states grow cereals as the major crops with a share of 68.95 per cent in the Gross Cropped Area (GCA), followed by commercial crops (9.09%), horticultural and fodder crops (12.59%). Whereas, tur farmers across the states grow pulses as the major crops with a share of 35.74 per cent in the gross cropped area, followed by oilseed (30.56%), horticultural and fodder crops (15.56%), cereal crops (9.20%) and other oilseed crops (8.33%). This implies that location-specific and farm-based cropping patterns have to be evolved with a due consideration given to the vital determinants such as land, topography, water availability, intensity and duration of sunlight, labour availability, cash or credit, power source and market demand. Among different crops cultivated by sugarcane farm households, commercial crops account for a major share of 61.93 per cent in the gross cropped area, followed by cereals, oilseeds and horticulture and fodder crops, with a share of about 12 per cent each in the gross cropped area. Further, that the small landholding size does not discourage these farmers from growing perennial crops, an implication that landholding size is not a major determinant of the cropping pattern.

Maize-farmers across the states grow crops like paddy, maize and soybean. It is evident from the table that cereal crops account for as high as 86.91 per cent of the gross cropped area, followed by oilseeds (about 12%). Whereas, soybean farmers across the states grow crops like paddy, soybean and cotton as their major crops. Among them, oilseed crop alone accounts for as high as 76.94 per cent of the gross cropped area, followed by cotton as a commercial crop (18.82%) and the least (4.23%) in the case of cereal crops. Being risk averse is a good practice followed by soybean farmers from the view point of generating income from other sources such as dairy-farming and cultivation of commercial crops such as pulses, cotton and cereals. However, in the case of jute farmers, jute apart, other crops are grown only for their subsistence purpose. The cropping pattern is dominated by jute and paddy, accounting for around 74.79 per cent and 18.94 per cent of the gross cropped area, respectively. Further, jute farmers are found to have devoted about 6.27 per cent of the GCA to vegetable production (Tables 6, 7, 8 and 9).

**Table 6:** Impact of NCU on production and marketing of reference crops. (Quintals/acre). Source: Authors’ estimates using field survey data.

Particulars	Paddy		Tur		Jute		Maize		Sugarcane		Soybean	
	NCU	NU	NCU	NU	NCU	NU	NCU	NU	NCU	NU	NCU	**NU**
Main product yield (quintal)	22.52	20.90** (7.75)	3.5	2.62Ns (33.58)	8.86	8.60** (3.02)	25.25	23.38* (7.99)	539	513 ** (5.06)	5.32	3.86** (37.82)
By-product Yield (quintal)	32.41	31.59Ns (2.59)	2.38	1.93* (23.31)	3.10	3.10NS (0.00)	16.32	16.31NS (0.06)	0.8	0.8 NS (0.00)	7.98	7.18* (11.14)
Price of main product (Rs/ quintal)	1373	1365** (0.58)	8217	8418NS (− 2.38)	2044	2055NS (− 0.53)	1049	1076NS (− 2.50)	221	216 * (2.31)	3151	3595* (− 12.35)
Price of by-product (Rs/ quintal)	182	167* (8.98)	431	489** (− 11.86)	250	250NS (0.00)	152	145NS (4.82)	415	356 NS (16.57)	179	163* (9.81)
Value of main product (Rs)	31,740	29326NS (8.23)	29,645	22419NS (32.23)	18,110	17,673* (2.47)	26,487	25,157* (5.28)	119,231	110,912 * (7.50)	16,763	13,877** (20.80)
Value of by-product (Rs)	4094	3305NS (23.87)	1032	747** (38.15)	775	775NS (0.00)	2481	2365* (4.90)	343	297 NS (15.48)	1428	1170** (22.05)

**^&^* denotes significance level at 1 and 5%, respectively. Figures in parentheses indicate percentage change.

**Table 7:** Impact of NCU use on the component-wise cost of reference crops. (Value in Rs/acre). Source: Authors’ estimates using field survey data.

Particulars	Paddy		Tur		Jute		Maize		Sugarcane		**Soybean**	
	NCU	NU	NCU	NU	NCU	NU	NCU	NU	NCU	**NU**	**NCU**	**NU**
Cost of pest and disease control	1362	1453* (− 6.26)	1090	1022** (6.65)	127	123* (3.25)	268	344Ns (− 22.09)	501	409 Ns (22.49)	689	817* (− 15.66)
Cost of weed management	569	601** (− 5.32)	453	323** (40.24)	1211	1212Ns (− 0.08)	213	221Ns (− 3.61)	607	604 NS (0.49)	443	486* (− 8.84)
Cost of NCU / Normal Urea	510	511** (− 0.19)	279	233Ns (19.74)	243	292** (− 16.78)	848	807* (5.08)	1451	1714 ** (− 15.34)	110	114** (− 3.50)
Cost of other fertilizers	5666	5194* (9.08)	2011	1199Ns (67.72)	1201	1210Ns (− 0.74)	11,794	10,109* (16.66)	5900	5628 ** (4.83)	1215	1209NS (0.49)
Total Cost	8107	7759** (4.48)	3833	2192** (74.86)	2782	2837* (− 1.93)	13,123	11,481* (14.30)	8459	8355 * (1.24)	2457	2626** (− 6.43)

**^&^ * denotes significance level at 1 and 5%, respectively; Figures in parentheses indicate percentage change.

**Table 8:** Economic feasibility of NCU use for reference crops (partial budgeting framework) (Rs/acre). Source: Authors’ estimates using field survey data.

Particulars	Paddy	Tur	Jute	Maize	Sugarcane	Soybean
Added cost	739.33	1300	0	1685.87	585	1141
Reduced cost	226.61	3	62	83.67	149	16
Added return	2942.69	16,558	–	–	–	–
Reduced return	–	321	554	1964.83	5,749	3942
B:C ratio	4.28	10.21		1.21	10.11	3.46

**Table 9:** Crop-wise results of t-values two sample paired t-tests with unequal variance. Source: Authors’ estimates using field survey data.

Sl.no.	Variables	Paddy	Tur	Sugar-cane	Maize	Soybean	Jute
1	Cost of NCU	2.361	1.370	2.818	2.163	3.245	2.423
	Cost of NU						
2	Cost of other fertilizer of NCU	1.780	1.455	3.304	2.123	0.605	1.816
	Cost of other fertilizer of NU						
4	Cost of pest and disease management NCU	1.943	2.440	2.731	1.392	2.741	5.902
	Cost of pest and disease management NU						
5	Quantity of main product in NCU	2.691	0.208	3.178	3.156	2.496	5.777
	Quantity of main product in NU						
6	Value of main product NCU	0.970	0.226	2.785	2.145	3.136	5.770
	Value of main product NU						
7	Quantity of by product of NCU	1.364	1.666	1.084	1.325	2.035	1.174
	Quantity of by product of NU						
8	Value of by-product NCU	0.213	3.330	1.939	2.589	3.945	1.298
	Value of by-product NU						
9	Price of main product of NCU users	3.301	1.47	2.142	1.891	2.658	1.031
	Price of main product of NU users						
10	Price of by-product of NCU users	2.112	2.473	0.742	1.458	3.021	0.847
	Price of by-product of NU users						
11	Total cost of NCU users	2.274	2.556	2.981	3.981	4.120	2.514
	Total cost of NU users						

### Impact of NCU on production and marketing of reference crops

During the reference period (Kharif 2015), both NU and NCU were available in the market across the study area before the government made mandatory the production (100%) and distribution of NCU throughout the country. Therefore, an effort was made by the study to compare the impact of NU and NCU on the production and productivity of reference crops across states in India. The details of the impact of NCU on production and marketing of reference crops are presented in Tables 6 and 9. A perusal of the table reveals that out of the sample crops, the average main product yield of soybean is more in the case of NCU users (5.32 quintals/acre), as compared to NU users (3.86 quintals/acre), accounting for a statistically significant increase in the yield level at 37.82 per cent, followed by tur (33.68%) and maize (7.99%). This is due to the presence of neem content in urea, which slows down the release of nitrogen and as a result, nitrogen (N) is available to plants for a longer period, as compared to NU and also concomitantly reduces the frequency of application and consumption of urea fertilizer. These results also conform to the study findings of^26–30^, who found a significant increase in grain yield of rice based on their successive field experiments. Similarly, in terms of by-product yield, the increase in yield amounts to 23.31 per cent, as compared to that of tur crop in the context of NU application, followed by soybean (11.14%). This increase in yield is found to be statistically significant. These findings also stand indicated by the study findings of^17,18,28,31^, who found that application of NCU following the site-specific nutrient management principles had led to crop production of higher or similar levels as observed with untreated urea, but with lower fertilizer application rates.

With regard to paddy and sugarcane, the prices of the main product appear to be relatively the same with regard to NCU and NU. The per cent change over post NCU application in place of NU varies within two per cent, whereas, in respect of tur, jute, maize and soybean crops, the prices seem to have decreased to the tune of 2.38, 0.53, 2.50 and 12.35 per cent, respectively, which could be due to market imperfections. Similarly, in the case of by-product price, the per cent change in respect of NCU, as compared to NU, amounts to 16.57 per cent in the case of sugarcane, followed by soybean (9.81%) and paddy (8.98%). The increase in the price of sugarcane by-product from Rs.356/ bundle (without NCU) to Rs. 415/ bundle (with NCU) might be attributed to the application of NCU, in addition to many other factors. Further, a majority of the farmers also have reported an increase in the quality of the main product and by-product yields, post NCU application. With respect to statistical significance, most of the prices of reference crops appear to be in-significant. Depending upon the prices of both the main product and by-product, the value of the main product and by-product of tur crop shows an increase of 32.23 per cent and 38.15 per cent, respectively, post the adoption of NCU in place of NU at the aggregate level, and is found statistically significant at one per cent level for the values of main product and in-significant for by-product.

### Impact of NCU use on the component-wise cost of reference crops

The details of the impact of NCU on the input costs of reference crops across the sample states are presented in Tables 7 and 9. To assess the impact of NCU usage on input costs, parameters such as the cost of pest and disease control, the cost of weed management, the cost of NCU or NU, and the cost of other fertilizers were considered. Tables 7 and 9 reveal a comparative picture of the input costs of NCU and NU using farmers. A perusal of the table reveals that the total cost of the selected inputs has increased for NCU users (Rs.3,833/acre), as compared to NU users (Rs.2,192/acre), to the extent of 74.86 per cent in respect of tur/pigeonpea crop, (Tur is a comman name of redgram and also commonly known as Pigeonpea (Arhar). The Botanical Name of Redgram/Tur is *Cajanus cajana* (L.) Millsp. followed by maize (14.30%) and paddy (4.48%), while in contrast, crops like jute and soybean show a decline in per cent change, at the aggregate level. Whereas the cost illustrates a decreasing scenario in respect of all the parameters, excepting the cost of other fertilizers for paddy and soybean farmers, respectively. An increasing trend in the cost of pest and diseases control, weed management, and other fertilizers can be seen for tur (6.65%, 40.24%, 67.72%) and sugarcane (22.49%, 0.49%, 4.83%), respectively. In terms of disparity, the decreasing trend in the cost of NCU/ NU works out to 0.19, 16.78, 15.34, and 3.50 per cent for paddy, jute, sugarcane and soybean, respectively. These results also conform to the study findings of^32–35^ who found an importance of aromatic rice in view, coated-urea materials and their effects on rice yields, nitrogen (N), and Zn content in different parts and input economics are evaluated.

### Economic feasibility of NCU use for reference crops using partial budgeting technique

An economic feasibility analysis of NCU use, often with and without NCU, has been used for identifying and assessing the costs and benefits as part of an evaluation of the current situation, more meaningfully. The difference between the costs and benefits is the net incremental benefit arising from NCU usage. However, a before and after approach has not been used in this framework on account of changes in production that would have occurred due to regular developments, along with NCU usage. While assessing the benefits and costs of NCU usage, only incremental net benefits need to be considered, with the reduced benefits treated as costs. The benefits foregone need to be taken as a cost component of NCU usage. Thereby, only incremental value could be attributed to NCU. Hence, a partial budget technique has been used for assessing the incremental income based on a small change in farm business post NCU application. In the present article, a partial budgeting framework has been estimated for variables such as additional income, reduced costs, reduced income and additional costs, following a small change in NCU use vis-a-vis NU. The budget indicates whether the change has increased/ decreased/ no change in the net income with the adoption of NCU. Also, the partial budget compares both the positive and negative effects of a change with NCU use in relation to NU, or an incremental income accruing from reference crops.

The impact of NCU, based on a partial budgeting technique, considering added and reduced costs with NCU application for reference crops is presented in Table 8. It can be seen from the table that there is a positive impact of the economic feasibility of NCU use on reference crops. The variables considered for estimating a partial budgeting framework in the study include the cost of pest and disease control, cost of weed management, cost of NCU/NU, and the cost of other fertilizers. In the table, only cost and returns are highlighted. At the aggregate level, the added costs with NCU application appear to be as high as Rs. 1,685.87 per acre in the case of maize, followed by tur (Rs. 1,300/acre) and soybean (Rs. 1,141/acre), respectively.

It is exceptional to note that the reduced returns are highest in the case of sugarcane (Rs. 5,749/acre), followed by soybean (Rs. 3,942/acre) and maize (Rs. 1,965/acre). Instead, added returns both in terms of the main product and by-product yields are noticed in respect of tur (Rs. 16,558 per acre) and paddy producers (Rs. 2,943/acre) only because of the adoption of NCU in place of NU. Whereas, reduced costs with NCU application are observed for almost all the sample crops with varying amounts. However, the reduced costs are found to be highest in the case of paddy (Rs. 227/acre), followed by sugarcane (Rs. 149/acre) and maize (Rs. 84/acre). This is the positive impact of NCU adoption in lieu of NU, in addition to other favourable factors. Using the same information, the benefit–cost ratio has been arrived at and presented in the same table. It is very much interesting to note that the BC ratio is more than 10 in respect of tur and sugarcane crops, meaning that, for every one rupee of investment on NCU application, there has been a rise in returns to the extent of Rs. 10. As regards paddy and soybean, the ratio works out to more than three per cent, while it is least (< 1%) in the case of maize. These results indicate that the application of NCU has had a positive impact in terms of both increased yield and income due to reduced costs for the farmers.

### Results of the logistic regression

The results of a logistic regression model are presented in Table 10. The results show that the age of household heads is statistically significant and negatively influence the probability of households being neem coated urea users. On the contrary, the probability of being a neem coated urea user is higher among farmers having more experience in farming profession with higher chances of adopting neem coated urea, as reflected by the ratio of the area under selected crops to the net operated area. The coefficient is also found highly significant for the ratio of neem coated urea cost to the total fertilizer cost, and net income earned from selected crops. Education and net operated area have no significant role in explaining the neem coated urea adoption.

**Table 10:** Results of logistic regression model for neem coated urea farmers at all India. Source: Authors’ estimates using field survey data.

SI. no.	Variables	Coefficient
1	Age (year)	− 0.062** (0.019)
2	Education (year of schooling)	− 0.026 (0.269)
3	Net operated area (acres)	− 0.086 (0.328)
4	Ratio of selected crop area to net operated area (%)	3.908** (0.423)
5	Experience in farming (year)	0.089* (0.043)
6	Ratio of neem coated urea cost to total fertilizer cost (%)	2.604** (0.441)
7	Net income (Rs)	3.501** (0.542)
	Constant	0.443
	Number of observation	1200
	LR Chi^2^	59.723*
	Prob > Chi^2^	0.000
	Pseudo R^2^	0.187

**^&^*Denotes significance level at 1 and 5%, respectively. Figures in parentheses indicate standard error.

### Impact of neem coated urea using propensity score matching

The impact of NCU and production and productivity of crops using propensity score matching technique is presented in Table 11. The balancing test for the individual covariates for NNM, KBM and CM shows that the bias for all covariates is less than 20% post matching, which is desirable. Table 12 reveals the appropriateness of the model, as reflected in the impressive Pseudo R^2^ and Correlation. The coefficient of Pseudo R^2^ shows a significant, decline to 1.5, 1.2 and 1.0%, respectively for NNM, KBM and CM after matching, which was found 18% before matching. The p-value is also not significant after matching as reflected in 0.567, 0.698, and 0.788, respectively. The MASB, which was 29% before matching, shows a decrease to 8.51, 9.23 and 7.24%, respectively for NNM, KBM and CM after matching. The coefficient of a low Pseudo R^2^, non significant p-values of the likelihood ratio test, low standardized biases and high reduction in the total bias after matching indicate that the specification of propensity score matching is impressive in terms of balancing the distribution of covariates across NCU and NU users.

**Table 11:** Propensity score matching by balancing test of individual covariates. Source: Authors’ estimates using field survey data.

Si. No.	Variables	Percentage of bias					
		Unmatched			Matched		
		Nearest neighbour	Kernel	Calliper	Nearest neighbour	Kernel	Calliper
1	Age	− 13.11	− 13.11	− 13.11	16.10	13.61	13.32
2	Education	1.31	1.32	1.32	0.81	− 4.84	3.93
3	Net operated Area (Landholdings)	14.50	14.50	14.50	8.23	13.22	7
4	Ratio of selected crops area to net operated area	80.20	80.20	80.20	7.50	8	4.51
5	Experience in farming	17	17	17	8.94	6.24	9
6	Ratio of neem coated urea cost to total fertilizer cost (%)	99.20	99.20	99.20	5.62	6	2.93
7	Net Income (Rs)	100.10	100.10	100.10	8.25	9	5.24

**Table 12:** Summary of model for balancing test of neem coated urea. Source: Authors’ estimates using field survey data.

Test	Before matching	After matching		
		Nearest neighbour	Kernel	Calliper
Pseudo R^2^	0.187	0.015	0.012	0.010
LR^2^ (P-value)	59.723 (0.000) **	5.61 (0.567)	4.82 (0.698)	4.02 (0.788)
Mean standardized bias	29.01	8.51	9.23	7.24
Total bias reduction (%)	–	75.33	72.87	77.03

*Denotes significance level at 1%.

### Diversion of urea for other than agricultural purposes

Based on the study, what can be inferred is that most of the farmers tend to use normal urea in smaller proportions as feed to cattle and fishes, mixed with milk for enhancing the fat content, and distilling local alcohol etc. However, the usage of urea has been completely stopped, post the introduction of NCU.

## Conclusions

### Conceptual contribution

The results based on extensive field trials indicate that, NCU is agronomical superior to normal urea. Various positive benefits associated with NCU use include slow release of N; by the improvement in uptake of N as well as P and K to a considerable extent; reduction in environmental hazards etc. Recognizing these benefits, the Union Government of India has made mandatory the production and distribution of NCU (100%) across the country from May 2015. The aim of the policy is to control the excessive use of urea in agriculture, besides preventing the diversion of subsidized urea towards industrial purposes.

### Practical implication

The study reveals that both the main product and by-product yield levels of all the reference crops have increased and that farmers are able to reap the positive externalities of NCU in terms of increased outputs, reduced costs (in terms of pest and disease control), thereby increased returns. Further, the coefficient of a low Pseudo R^2^, non significant p-values of the likelihood ratio test, low standardized biases and a high reduction in the total bias after matching indicate that the specification of propensity score matching is impressive in terms of balancing the distribution of covariates across neem coated urea and normal urea users. More importantly, these results, point to the effectiveness of the policy in terms of a decline observed in the cost of urea consumption, to an extent, increased returns and the stoppage of diversion of urea to industrial purposes post the introduction of NCU.

### Limitations and future research

With regarding to the limitations of the study, the benefits observed by in the study might not be related to NCU usage alone, as some other favourable factors might have contributed to the same as well; it is very difficult to realize all the potential benefits associated, with NCU use within a limited period, because the study was conducted immediately after the implementation of NCU policy in the country (the study was limited to one season i.e., Kharif 2015); there was lack of timely availability of NCU during Kharif 2015 due to delay involved in the policy implementation. Further, there was a stock of normal urea available in the market during the study period and hence, a few farmers might have applied both NCU and NU; the usage of NCU relative to NU has not been very impressive, which might be due to the ignorance of farmers about the potential benefits of NCU over NU and its application, as the mandatory production of NCU was commissioned for the first time in the country.

The areas of future research are: this study was a baseline survey and hence, can be repeated after a few years in order to get a better picture of NCU use; the results of the study can also be validated by experimental research; various concentrations of neem oil coated urea and other major nutrients and their impacts may help get optimum benefits from NCU use.

## Data Availability

The datasets generated and/or analysed during the current study are available in the project report ['Impact of Neem Coated Urea on production, Productivity and Soil Health in India'] repository [http://www.isec.ac.in/NCU-final%20consolidated%20report%2017082017-revised-print.pdf].
